# The changes and its significance of peripheral blood NK cells in patients with tuberculous meningitis

**DOI:** 10.3389/fmicb.2024.1344162

**Published:** 2024-02-29

**Authors:** Jie Mi, Yinping Liu, Yong Xue, Wenna Sun, Yan Liang, Jianqin Liang, Huiru An, Xueqiong Wu

**Affiliations:** ^1^Beijing Key Laboratory of New Techniques of Tuberculosis Diagnosis and Treatment, Institute of Tuberculosis Research, Senior Department of Tuberculosis, The 8th Medical Center of PLA General Hospital, Beijing, China; ^2^Department of Tuberculosis, Senior Department of Tuberculosis, The 8th Medical Center of PLA General Hospital, Beijing, China

**Keywords:** tuberculous meningitis, lymphocyte subsets, NK cells, peripheral blood, disease severity, IGRA, antibody

## Abstract

**Objective:**

Tuberculous meningitis (TBM) is the most severe form of tuberculosis (TB). The purpose of this study was to explore the relationship between the number of natural killer (NK) cells and adaptive immune status, and disease severity in TBM patients.

**Methods:**

We conducted a retrospective study on 244 TB patients and 146 healthy control subjects in the 8th Medical Center of the PLA General Hospital from March 2018 and August 2023.

**Results:**

The absolute count of NK cells in the peripheral blood of TBM patients was significantly lower than that in normal controls (NC), latent tuberculosis infection (LTBI), and non-severe TB (NSTB) patients (*p* < 0.05). The proportion of TBM patients (48.7%) with a lower absolute count of NK cells than the normal reference value was significantly higher than that in NC (5.2%) and LTBI groups (4.0%) (*p* < 0.05), and slightly higher than that in NSTB group (36.0%) (*p* > 0.05). The absolute counts of lymphocyte subsets in TBM combined with other active TB group, etiology (+) group, IGRA (−) group, and antibody (+) group were lower than that in simple TBM group, etiology (−) group, IGRA (+) group, and antibody (−) group, respectively. The CD3^+^ T, NK, and B cells in BMRC-stage III TBM patients were significantly lower than those in stage I and stage II patients (*p* < 0.05). The counts of CD3^+^ T, CD4^+^ T, and B cells in the etiology (+) group were significantly lower than those in the etiology (−) group (*p* < 0.05).

**Conclusion:**

The absolute counts of lymphocyte subsets in the peripheral blood of TBM patients were significantly decreased, especially in NK cells. The reduction of these immune cells was closely related to the disease severity and had a certain correlation with cellular and humoral immune responses. This study helps to better understand the immune mechanism of TBM and provides reliable indicators for evaluating the immune status of TBM patients in clinical practice.

## 1 Introduction

Tuberculosis (TB) is a chronic respiratory infectious disease caused by *Mycobacterium tuberculosis* (*Mtb*). According to the latest World Health Organization (WHO) report, there were approximately 10.6 million new cases and 1.3 million deaths worldwide in 2022 (WHO, 2023). Worldwide, it is estimated that one-fourth of the population is infected with *Mtb*, and 5–10% of those infected are at risk of further progression to active TB (ATB) (Paton et al., 2019). *Mtb* can infect any organ system in the body with pulmonary TB (PTB) being its most common presentation, and the rest as extrapulmonary TB (EPTB). Among them, tuberculous meningitis (TBM) represents the most severe form of TB, with high morbidity and mortality, and with more than 100,000 new cases reported by the WHO every year (Poh et al., 2021; Li et al., 2022).

TB is a typical bacterial infectious disease and also an immune-related disease, which is affected by a variety of factors, such as bacterial load and host immunity, especially local anti-TB immunity of the lung. After *Mtb* invasion, the initial acute immune response against *Mtb* is initiated by the innate immune system of the body to eliminate the pathogen. Innate immune cells include macrophages, natural killer (NK) cells, dendritic cells, etc., which represent important components of the innate immune defense mechanisms. They mainly play the role of phagocytosis and antigen presentation, clearing *Mtb* in macrophages and producing cytotoxic effects to destroy lysed target cells (Garand et al., 2018; Choreño-Parra et al., 2020, 2021). Subsequently, the adaptive immune responses are activated, in which the cellular immune responses mediated by T cells (including CD4^+^ and CD8^+^ T cells) and their production of Th1 cytokines (such as IFN-γ and IL-2) play a major anti-TB role, and the humoral immune responses mediated by B cells and antibodies exert an adjuvant effect (Mi et al., 2021; Wu Xueqiong, 2022). Therefore, the activation of the body’s immune system and inflammatory responses play a critical role in the immune pathogenesis of diseases. Studies have found that these changes in immune responses were closely related to the occurrence and development of this disease (An et al., 2022). However, most current research has only been conducted in ATB patients, latent TB infection (LTBI), or tuberculous pleurisy, and few studies have reported the clinical immunological characteristics of TBM patients.

Presently, the pathogenesis of TBM is still insufficiently understood. Researchers in the last century have found that *Mtb* infected the central nervous system long before TBM patients showed symptoms, but the associated clinical symptoms did not manifest immediately in the brain (Rich, 1933). NK cells are a unique group of granular lymphocytes of the innate immune system with innate cytotoxicity and immune regulatory ability, playing a crucial role in the early stages of infection. NK cells can present signals to infected dendritic cells and macrophages to assist the body in clearing *Mtb* (Esin et al., 2013; Choreño Parra et al., 2017). NK cells can also exert classical cytotoxic effects by secreting perforin and granzymes to clear mycobacterium and *Mtb*-infected host cells (Brill et al., 2001; Lu et al., 2014). Additionally, NK cells can secrete cytokines to regulate the immune response, such as IFN-γ, TNF-α, and IL-22 (Feng et al., 2006; Zhang et al., 2006). However, little work has been reported on the phenotypic changes of NK cells in TBM patients. Only several studies found that the NK cells in the peripheral blood of TBM patients were lower than those in healthy controls, and CD56^bright^CD16^−^ NK cells were significantly lower than those in LTBI population and PTB patients, while neutrophils and classical monocytes were significantly higher, but the number of TBM patients enrolled was limited (van Laarhoven et al., 2019; Choreño-Parra et al., 2021; Shridhar et al., 2022). There were no studies on the mechanisms involved in the immune response after *Mtb* infection in TBM patients. Therefore, the study of the number of NK cells and the adaptive immune status of TBM patients can provide new insights and ideas to elucidate the pathogenesis of TBM.

Currently, flow cytometry (FCM) is commonly used to analyze peripheral blood lymphocyte subsets in TB patients to assess immune status, immune function, and immune balance. In this study, we analyzed the changes in the percentages and absolute counts of CD3^+^, CD4^+^, CD8^+^, NK, NKT, and B lymphocytes in peripheral blood of TBM patients, non-severe TB (NSTB) patients, LTBI persons, and normal controls (NC) through FCM. In addition, the relationship between the number of immune cells in TBM patients and the disease severity, bacterial load in the body, cytokines, and antibody levels, as well as the changes in NK cells in the TBM population were emphatically analyzed, providing a reference basis for further understanding of the immune mechanism of TBM and clinical evaluation of the immune status of TBM patients.

## 2 Materials and methods

### 2.1 Subjects

This study was approved by the Ethics Committee of the 8th Medical Center of the PLA General Hospital (approval number: 309201904081530). A total of 244 hospitalized TB patients from the 8th Medical Center of the PLA General Hospital from March 2018 and August 2023 were included in the present study. The diagnosis of TB was carried out and guided according to the People’s Republic of China’s Health Standards “Diagnosis for Pulmonary Tuberculosis (WS 288–2017)” and “Classification of Tuberculosis (WS 196–2017)” issued by the National Health and Family Planning Commission of the People’s Republic of China: (1) TB-related symptoms included at least one of cough, expectoration, hemoptysis, fever, loss of appetite, fatigue, night sweats, etc.; (2) Chest X-ray or CT indicated abnormal lesions; (3) Histopathological examination revealed tuberculous lesions; (4) The smear, culture, and/or molecular biology detection on clinical samples exhibited positive; (5) Anti-TB treatment was effective and excludes other non-TB diseases.

1. TBM group: 119 cases (73 males and 46 females, aged 18–79 years) diagnosed with TBM were further divided into the following subgroups based on clinical parameters: according to clinical diagnosis, the cases were divided into a simple TBM group (*n* = 40) and a TBM combined with other ATB group (TBM-ATB, *n* = 79); According to the duration of treatment, the cases were divided into an initial treatment group (In-TBM, *n* = 37) and a re-treatment group (Re-TBM, *n* = 82); According to the results of smear, culture, and/or molecular biological test on clinical samples [cerebrospinal fluid (CSF)/sputum], the cases were divided into an etiology-positive group [EG (+), *n* = 17] and an etiology-negative group [EG (−), *n* = 58]; According to the modified British Medical Research Council (BMRC) grading system and the Glasgow Coma Scale, the cases were classified into stage I (*n* = 87), stage II (*n* = 22), and stage III (*n* = 10) (Alarcón et al., 2013; Poh et al., 2021); According to the results of interferon-gamma release assay (IGRA), the cases were divided into an IGRA (+) group (*n* = 55) and an IGRA (−) group (*n* = 33); According to the results of antibody IgG detection, the cases were divided into an antibody-positive group [Ab (+), (*n* = 46)] and an antibody-negative group [Ab (−), (*n* = 39)]. The data were collected and reviewed retrospectively based on clinical records, and not all patients have been undergoing etiological and immunological testing, resulting in fewer grouped cases than the total number of cases.
2. NSTB group: 125 cases (81 males and 44 females aged 18–97 years), including PTB, tuberculous pleurisy, bronchial TB, and other TB patients, excluding PTB complicated by massive hemoptysis, PTB complicated by respiratory failure, hematogenous disseminated TB, TBM, and other severe TB patients.
3. LTBI group: 50 cases (28 males and 22 females, aged 19–65 years) who were defined as close contacts of active PTB patients in the same period, with positive IGRA, no clinical symptoms of TB, and no abnormalities in imaging examination.
4. NC group: 96 cases (50 males and 46 females, aged 18–70 years), with negative IGRA, no history of TB, no clinical manifestations of TB, and no abnormality in imaging examination.

Exclusion criteria were as follows: having autoimmune diseases; severe liver and kidney dysfunction; HIV positive; and during pregnancy or lactation.

### 2.2 Etiological detection methods

CSF/sputum was obtained from all TBM patients and detected using smear, conventional culture, and/or molecular biology methods. Any participant with at least one positive test result was defined as a TB patient with positive etiology. Ziehl-Neelsen (ZN) acid-fast staining and BACTEC MGIT 960 system (BD Biosciences, Franklin Lake, United States) were used for smear and culture, respectively. Real-time qPCR (CapitalBio Corporation, China) and/or Xpert MTB/RIF (Cepheid, Sunnyvale, United States) were used to detect *Mtb* DNA in specimens.

### 2.3 IGRA test methods

A total of 3–4 mL venous blood from the subjects was collected and allowed to store at room temperature for a maximum of 6 h before analysis. Peripheral blood mononuclear cells (PBMCs) were isolated by Ficoll-Paque^PLUS^ (GE Healthcare Life Sciences) gradient centrifugation. Then, IGRA tests [including ELISPOT (Mabtech, Sweden) and ELISA (Wantai Biological Pharmacy Enterprise, Beijing, China) kits] were used to quantitatively detect the specific T-cell immune response of human whole blood samples stimulated by *Mtb*-specific antigen *in vitro*, which is used to assist the diagnosis of *Mtb* infection.

### 2.4 Antibody test method

A total of 3–5 mL peripheral venous blood from the subjects was collected, and serum was separated by centrifugation. The detection of *Mtb* IgG antibody in human serum samples was performed *in vitro* using the *Mtb* IgG antibody detection kits (colloidal gold method) (Beijing Zhongjian Antai Diagnostic Technology Co., Ltd.; Shanghai Aopu Biopharmaceutical Co., Ltd., China).

### 2.5 BMRC grading system

According to the BMRC grading criteria, we divided TBM patients into stage I, stage II, and stage III based on their mental status and neurological signs. Stage I: has alertness and directional ability, without any signs of neurological localization. Stage II: conscious but lack of concentration, blurred consciousness, and drowsiness, with possible mild focal signs such as cranial nerve palsy or mild hemiplegia (Glasgow Coma Scale score 11–15) (Table 1). Stage III: in advanced stages of the disease, accompanied by delirium, lethargy, coma, epileptic seizures, multiple cranial nerve palsy, and/or hemiplegia with equal involvement of the upper and lower limbs (Glasgow Coma Scale score ≤ 10) (Table 1) (Teasdale and Jennett, 1974; Holmes et al., 2005; Tuberculous meningitis Professional Committee, 2020; Glasgow Coma Scale, n.d.).

**Table 1:** Glasgow coma scale and pediatric Glasgow coma scale.

Sign	Glasgow coma scale (Teasdale and Jennett, 1974)	Pediatric Glasgow coma scale (Holmes et al., 2005)	Score
Eye opening	Spontaneous	Spontaneous	4
	To command	To sound	3
	To pain	To pain	2
	None	None	1
Verbal response	Oriented	Age-appropriate vocalization, smile, or orientation to sound; interacts (coos, babbles); follows objects	5
	Confused, disoriented	Cries, irritable	4
	Inappropriate words	Cries to pain	3
	Incomprehensible sounds	Moans to pain	2
	None	None	1
Motor response	Obeys commands	Spontaneous movements (obeys verbal command)	6
	Localizes pain	Withdraws to touch (localizes pain)	5
	Withdraws	Withdraws to pain	4
	Abnormal flexion to pain	Abnormal flexion to pain (decorticate posture)	3
	Abnormal extension to pain	Abnormal extension to pain (decorticate posture)	2
	None	None	1
Best total score			15

### 2.6 Flow cytometry

A total of 4 mL fasting venous blood samples from subjects were collected in heparin anticoagulant tubes in the early morning and stored at room temperature. 50 μL whole blood was added in a test tube, followed by adding 10 μL BD Multitest 6-Color TBNK Reagent (BD Biosciences, United States) and mixed gently with vortex. The mixture was incubated at room temperature in the dark for 15 min. Then 450 μL fluorescence-activated cell sorter (FACS) lysing solution (BD Biosciences, United States) was added and mixed well, and further incubated at room temperature in the dark for 15 min. The absolute numbers and percentages of peripheral blood T lymphocyte subsets were determined using the FACS Aria II flow cytometer (BD Bioscience, United States). At least 5,000 lymphocytes were collected for each specimen and analyzed by FACS DIVA software. According to CD45-SSC, white blood cells were divided into lymphocytes, monocytes, and neutrophils. Lymphocytes were defined as follows: CD3^+^ T cells were defined as CD3^+^, CD4^+^ T cells were defined as CD3^+^CD4^+^CD8^−^, CD8^+^ T cells were defined as CD3^+^CD4^−^CD8^+^, NK cells were defined as CD3^−^CD16^+^ or/and CD56^+^, NKT cells were defined as CD3^+^CD16^+^ or/and CD56^+^, and B cells were defined as CD3^−^CD19^+^. The absolute numbers and percentages of CD3^+^ T, CD4^+^ T, CD8^+^ T, NK, NKT, and B cells were calculated, respectively. We used previously published normal values of lymphocyte subsets in Chinese Han people as a reference (Xu et al., 2021).

### 2.7 Statistical analysis

All data were statistically analyzed using GraphPad Prism 8.0 software (GraphPad Software, Inc.). The comparison between the two groups was conducted with the Wilcoxon rank sum test, multiple group comparisons were performed using the Kruskal-Wallis rank sum test. Chi-squared test was used to compare categorical data between groups. Differences less than 0.05 (*p* < 0.05) were considered statistically significant.

## 3 Results

### 3.1 Demographic and clinical data of the study population

A total of 390 cases were included in this investigation. There were no statistical differences in age and gender among the NC, LTBI, NSTB, and TBM groups (Table 2).

**Table 2:** Comparison of patient clinical data for each group.

Parameters	NC (*n* = 96)	LTBI (*n* = 50)	NSTB (*n* = 125)	TBM (*n* = 119)	*χ*^2^	*p* value
Age, years, median (range)	35 (18–70)	47.5 (19–65)	44 (18–97)	34 (18–79)		
Age (%)					10.636	0.1006
<25	18 (18.8%)	8 (16.0%)	27 (21.6%)	27 (22.7%)		
25–59	63 (65.6%)	34 (68.0%)	66 (52.8%)	76 (63.9%)		
≥60	15 (15.6%)	8 (16.0%)	32 (25.6%)	16 (13.4%)		
Gender (%)					4.070	0.2540
Male	50 (52.1%)	28 (56.0%)	81 (64.8%)	73 (61.3%)		
Female	46 (47.9%)	22 (44.0%)	44 (35.2%)	46 (38.7%)		

### 3.2 Comparison of lymphocyte subset levels in peripheral blood among all groups

**Figure 1:**
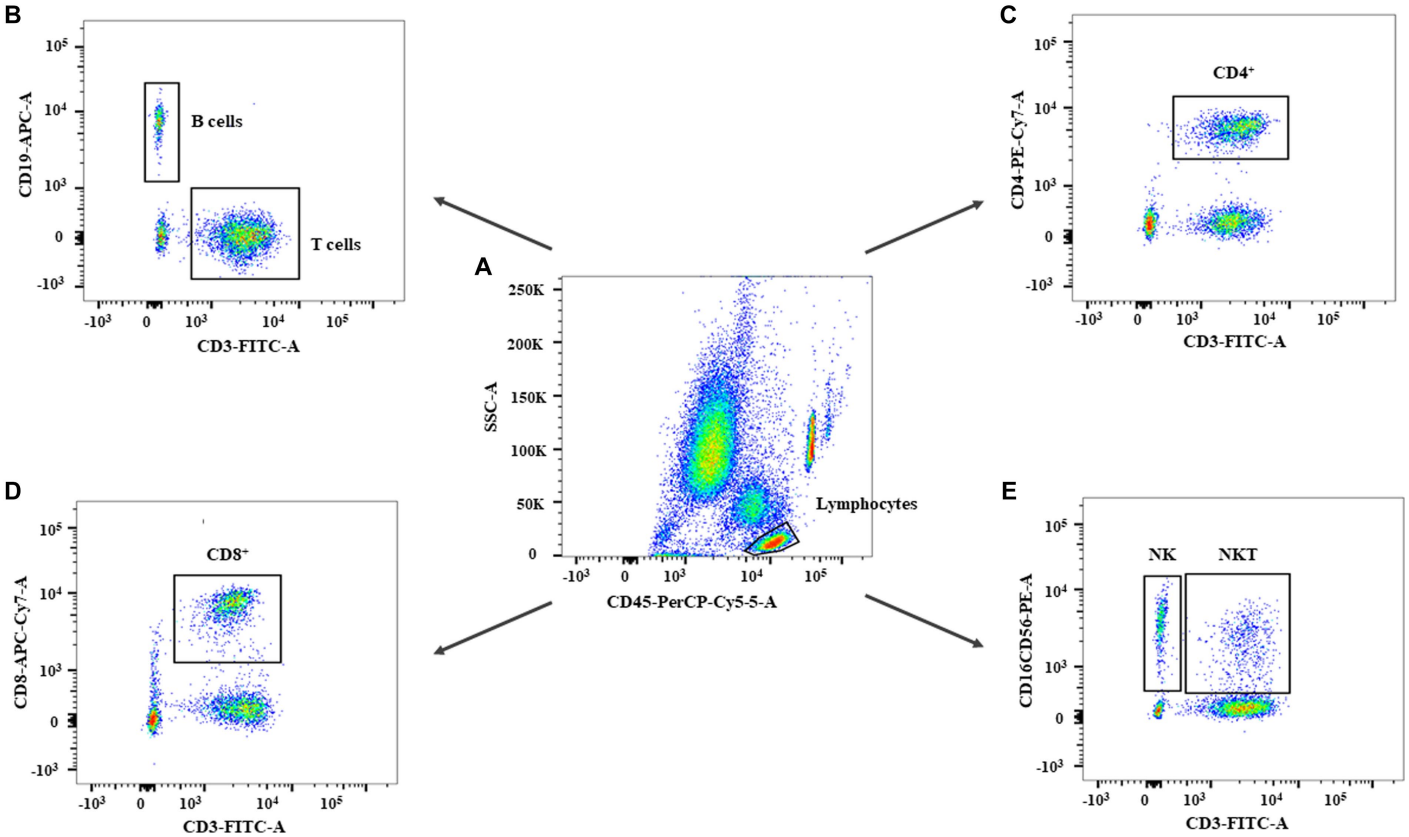
The flow cytometry gating strategy of subjects in this study. The gating strategy for leukocytes **(A)**, B cells and T cells **(B)**, CD4^+^ T cells **(C)**, CD8^+^ T cells **(D)**, NK and NKT cells **(E)**.

**Figure 2:**
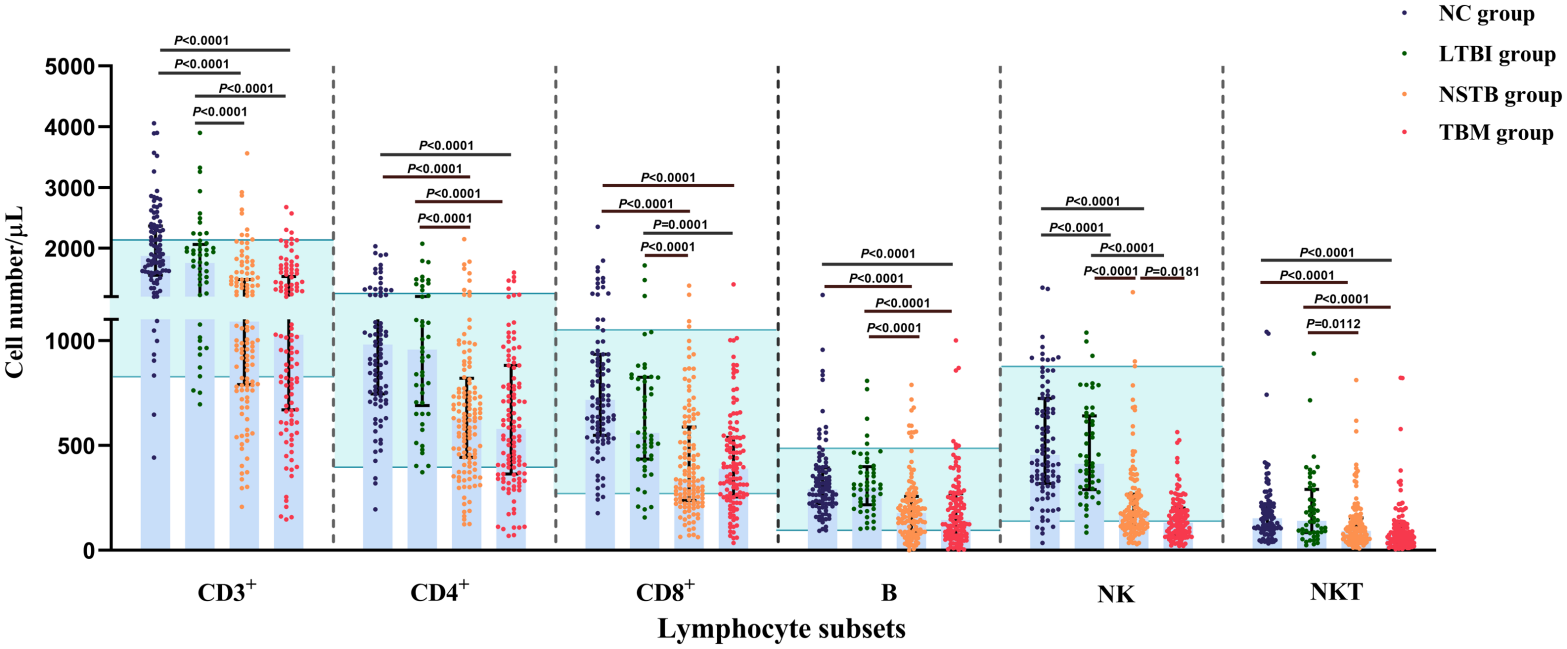
Comparison of absolute counts of peripheral blood lymphocyte subsets among NC, LTBI, NSTB, and TBM groups. NC: *n* = 96, LTBI: *n* = 50, NSTB: *n* = 125, TBM: *n* = 119. The absolute counts of lymphocyte subsets in peripheral blood were measured by flow cytometry. The error bars represent the median and interquartile range. The green shaded region represents the reference ranges in the Chinses population (Xu et al., 2021). The comparison between the two groups was conducted with the Wilcoxon rank sum test, multigroup comparisons were performed using the Kruskal-Wallis rank sum test. NC, normal population; LTBI, latent tuberculosis infection; NSTB, non-severe tuberculosis; TBM, tuberculous meningitis.

The gating strategy of this study is shown in Figure 1. In the percentage of lymphocyte subsets in each group, the percentages of CD3^+^ and CD4^+^ T cells in the TBM and NSTB groups were significantly higher than that in the NC group and LTBI group (*p* < 0.05), while the percentages of NK cells in the TBM and NSTB groups were significantly lower than that in the NC group and LTBI group (*p* < 0.05) (Supplementary Table S1). The absolute counts of CD3^+^ T, CD4^+^ T, CD8^+^ T, NK, NKT, and B cells in the TBM group and NSTB group were significantly lower than those in the NC and LTBI group, with significant differences (*p* < 0.05) (shown in Figure 2). Among them, the absolute counts of all cell subsets in TBM group were decreased compared with those in the NSTB group, but only the differences in NK cells achieved statistical significance (*p* < 0.05).

**Figure 3:**
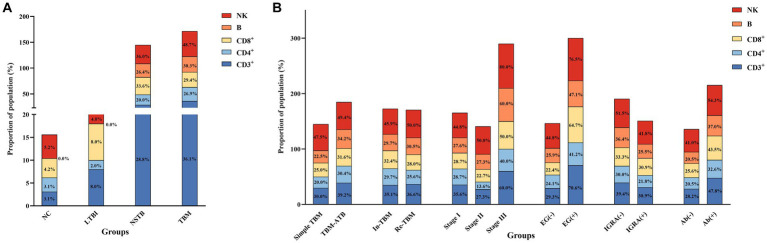
Proportion of the population in each group below the normal reference values for the absolute counts of lymphocyte subsets. **(A)** Comparison of population proportions among NC, LTBI, NSTB, and TBM groups. **(B)** Comparison of population proportions in TBM groups grouped according to different characteristics. The reference values for CD3^+^, CD4^+^, CD8^+^, B, and NK cells were 834 cells/μL, 395 cells/μL, 269 cells/μL, 92 cells/μL, and 136 cells/μL, respectively (Xu et al., 2021).

The proportion of the cases with lymphocyte subsets below the normal reference values in the NSTB and TBM groups was significantly higher than that in the NC and LTBI groups (*p* < 0.05) (shown in Figure 3A). Compared to the NSTB group, the TBM group showed an increasing trend in the proportion of the cases below normal reference values, but without significant differences.

The proportion of the cases with lymphocyte subsets below the normal reference values in the TBM-ATB, BMRC-Stage III, and EG (+) groups was significantly higher than that in the simple TBM, BMRC-stage I and II, and EG (−) groups, respectively. Among them, there was a significant difference (*p* < 0.05) between the BMRC-stage III group and BMRC-stage II group, and the proportion of the cases with CD3^+^ T, CD8^+^ T, and NK cells below the normal reference values in the EG (+) group was significantly higher than that in the EG (−) group (*p* < 0.05) (Figure 3B). The proportion of the cases with peripheral blood lymphocyte subsets below the normal reference values in the IGRA (−) group and Ab (+) group was higher than that in the IGRA (+) group and Ab (−) group, respectively, and the incidence of IGRA (−) and Ab (+) in the TBM patients tends to increase with the increase of the disease severity (Supplementary Table S2), but there was no significant difference (*p* > 0.05). And there was also no significant difference between the In-TBM group and the Re-TBM group (Figure 3B).

### 3.3 Relationship between absolute counts of peripheral blood lymphocyte subsets and disease severity, etiological examination, and immune responses in TBM cases

The relationship between absolute counts of peripheral blood lymphocyte subsets and disease severity, etiological examination, and immune responses in TBM groups is shown in Table 3.

**Table 3:** The relationship between absolute counts of peripheral blood lymphocyte subsets and disease severity, etiological examination results, and immune response in TBM population.

Groups	*n*	CD3^+^ (cells/μL) (median, range)	CD4^+^ (cells/μL) (median, range)	CD8^+^ (cells/μL) (median, range)	NK (cells/μL) (median, range)	NKT (cells/μL) (median, range)	B (cells/μL) (median, range)
TBM	119	1,027 (148–2,680)	579 (69–1,600)	389 (36–1,407)	132 (21–564)	73 (5–822)	144 (3–1,001)
Simple TBM	40	1,322 (148–2,573)	767 (73–1,600)	410 (61–1,001)	146 (21–440)	66 (15–821)	222 (14–1,001)
TBM-ATB	79	955 (158–2,680)	525 (69–1,525)	341 (36–1,407)	126 (21–564)	73 (5–822)	117 (3–857)^a^
**Treatment duration**							
In-TBM	37	1,243 (148–2,302)	585 (69–1,467)	386 (61–1,407)	137 (21–513)	91 (13–578)	158 (3–1,001)
Re-TBM	82	1,001 (158–2,680)	543 (102–1,600)	352 (36–1,002)	129 (21–564)	64 (5–822)	136 (4–870)
**BMRC**							
Stage I	87	1,025 (162–2,680)	579 (69–1,600)	379 (58–1,407)	146 (21–564)	75 (7–822)	144 (3–1,001)
Stage II	22	1,185 (158–1854)	620 (109–1,153)	466 (36–883)	129 (32–440)	51 (14–222)	202 (15–505)
Stage III	10	671 (148–1720)^b^^,^^c^	415 (73–917)	238 (61–610)	76 (40–166)^b^	108 (5–325)	60 (14–242)^b^^,^^c^
**CSF/sputum**							
EG (−)	58	1,124 (162–2,302)	650 (69–1,467)	396 (90–1,407)	146 (21–564)	84 (7–578)	153 (3–505)
EG (+)	17	671 (237–2,134)^d^	407 (102–1,335)^d^	254 (58–765)	103 (32–267)	53 (8–381)	95 (10–337)^d^
**Cellular immune**							
IGRA (−)	33	1,077 (158–2,573)	524 (109–1,600)	344 (36–1,407)	120 (32–513)	46 (14–824)	156 (44–1,001)
IGRA (+)	55	1,105 (237–2,259)	622 (102–1,467)	380 (111–1,011)	146 (52–525)	90 (11–578)^e^	131 (15–440)
**Humoral immune**							
Ab (−)	39	1,259 (162–2,259)	711 (69–1,467)	440 (90–865)	150 (21–564)	69 (11–578)	222 (5–505)
Ab (+)	46	886 (148–2,573)	441 (73–1,600)^f^	296 (36–1,011)	111 (32–314)	64 (5–821)	99 (4–1,001)^f^

TBM, Tuberculous meningitis; TBM-ATB, TBM combined with other ATB; In-TBM, Initial treatment TBM; Re-TBM, Re-treatment TBM; BMRC, British Medical Research Council; CSF, Cerebrospinal fluid; EG, Etiology; IGRA, Interferon-gamma release assay; AB, Antibody.

a *p* < 0.05 vs. simple TBM.

b *p* < 0.05 vs. BMRC-Stage I.

c *p* < 0.05 vs. BMRC-Stage II.

d *p* < 0.05 vs. EG (−).

e *p* < 0.05 vs. IGRA (−).

f *p* < 0.05 vs. Ab (−).

The counts of all lymphocyte subsets in TBM-ATB were lower than those in the simple TBM group, but the count of only B lymphocytes showed a significant difference (*p* < 0.05). The counts of lymphocyte subsets in the In-TBM group were higher than those in the Re-TBM, but with no significant difference (*p* > 0.05). In addition, there was no significant difference in the counts of all lymphocyte subsets between BMRC-stage II and stage I patients, while their counts in BMRC-stage III patients were significantly lower than those in stage I and stage II except for NKT cells, in which CD3^+^ T, NK, and B cells had significant differences (*p* < 0.05). Besides, the EG (+) group showed lower counts than those in the EG (−) group, with statistical significance in the counts of CD3^+^ T, CD4^+^ T, and B lymphocytes (*p* < 0.05). In the cellular immunity grouping, the number of CD3^+^ T, CD4^+^ T, CD8^+^ T, NK, and NKT cells in the IGRA (+) group was higher than those in IGRA (−) group, with only NKT cells having significant difference (*p* < 0.05), while the B cell count was lower than the IGRA (−) group without significant difference (*p* > 0.05). In the humoral immunity grouping, the absolute counts of lymphocyte subsets in the Ab (+) group were lower than those in the Ab (−) group, with significant differences in the counts of CD4^+^ T and B cells (*p* < 0.05).

## 4 Discussion

When *Mtb* invades the body, the host’s innate immune response is rapidly activated to play an early defense role and then initiates a specific cellular immune response leading to the formation of tuberculous granuloma. Cellular immunity is the main immune protection mechanism for TB, and TB will be developed when the human immune function becomes unbalanced or abnormal. TBM occurs if *Mtb* succeeds in crossing the blood–brain barrier through blood circulation or other means when the infection is not effectively controlled. The pathogenesis of TBM is still unclear, and it is known that T lymphocyte-mediated chronic inflammation is an important link in the body’s resistance to *Mtb*. The reduction of T lymphocytes and loss of their function are common pathological changes in the occurrence and development of TBM. NK cells, as important innate immune cells, can exert cytotoxicity in the initial stage of *Mtb* infection, kill extracellular *Mtb*, trigger the effector mechanisms of the macrophage and dendritic cell, and limit the intracellular growth of *Mtb*. In addition, NK cells can produce cytokines (such as IFN-γ, IL-12, etc.) and regulate the activation and function of *Mtb*-specific T cells (Esin and Batoni, 2015), thereby participating in the immune defense process of LTBI and active PTB (Choreño-Parra et al., 2021). However, there is currently limited research on NK cells in TBM, and their role in TBM is not yet fully understood.

Previous studies have shown that TB patients, including TBM and NSTB, were significantly immunocompromised, and their absolute counts of lymphocyte subsets were significantly lower than those of the NC and LTBI populations (Al Majid and Abba, 2008; Mi et al., 2021; Wu Xueqiong, 2022), which is consistent with the findings of our study. If CD3^+^ T, CD4^+^ T, CD8^+^ T, and NK cells are significantly reduced in PTB patients, it indicates that the body is prone to expand the range of lesions, appear cavities, and even spread outside the lungs to further develop into patients with EPTB including TBM (Guglielmetti et al., 2013). The study by Choreno Parra et al. demonstrated that NK cells were only activated during the active phase of *Mtb* infection and entered tissues (Choreño-Parra et al., 2021). Our study also found that compared with NSTB patients, the decrease of NK cells in TBM patients was particularly noticeable, indicating that the local innate immune responses of the lungs in TBM patients may be significantly attenuated, which causes the spread of *Mtb* in the lungs, thereby leading to the occurrence of TBM (Choreño-Parra et al., 2021). It is also possible that peripheral blood NK cells migrate to the central nervous system and produce pro-inflammatory cytokines, causing clinically common brain injury or other symptoms. Van Laarhoven et al. also found that the αβT, γδT, and NK cells in the blood of TBM patients were significantly reduced compared with PTB and healthy control groups, while the increased leucocyte activation and a predominance of αβT and NK cells in CSF were associated with better survival (van Laarhoven et al., 2019). Also, TBM patients have shown increased myeloid cell responses with diverse immune functions (van Laarhoven et al., 2019). In addition, flow cytometry confirmed the presence of αβT, γδT, B, NK, NKT, and MAIT cells in the CSF of TBM patients, with NK cells being the most abundant cell type besides αβT cells (Dieli et al., 1999; Simmons et al., 2005; van Laarhoven et al., 2019). The above literature studies have indirectly supported our findings that NK cells in TBM patients may be transferred from peripheral blood to CSF, but the number and functional changes of the transfers are still unknown. Therefore, we will simultaneously study the lymphocyte subsets in both CSF and peripheral blood of TBM patients to analyze the distribution, quantity, and function of lymphocyte subsets, especially NK cells in different clinical specimens of these patients, in order to discover more effective blood and CSF biomarkers and provide more reliable indices for future host-directed treatment of TBM.

At present, there are two methods for analyzing lymphocyte subsets in clinical practice. Among them, the relative counting method is to analyze the percentage of each subset of cells in the total number of lymphocytes, so the results are easily affected by changes in various cell subsets. The absolute counting method can understand the independent changes of various cell subsets. The change in the absolute count of one lymphocyte subset does not affect the changes in the absolute count of other cell subsets, but may affect the changes in the relative count of lymphocyte subsets (Morais-Papini et al., 2017). In this study, the percentage of NK cells in the TBM and NSTB groups significantly decreased, leading to significant increases in the percentages of CD3^+^ and CD4^+^ T cells, but the absolute counts of these three types of cells were significantly reduced. Guglielmetti et al. also found that the absolute count of all lymphocyte subsets in patients with severe TB decreased, but the percentage of all lymphocyte subsets may not change significantly, still in the normal range (Guglielmetti et al., 2013). Therefore, it is recommended to simultaneously detect and analyze the relative and absolute counts of lymphocyte subsets in TB patients, to objectively and accurately reflect the immune cell status of TB patients. Our findings provided further evidence that TBM was associated with a decrease in peripheral blood lymphocytes, which was also closely related to disease severity (Guglielmetti et al., 2013). In particular, the decreases of NK cells and B cells were closely associated with the degree of disease spread and bacterial load in TBM patients, which is consistent with the results of previous studies (Davoudi et al., 2008; Yang Ming et al., 2017), suggesting that severe TBM patients may have immune cell exhaustion and more severe cellular immunosuppression in the body.

In the process of adaptive immune against TB, effector CD4^+^ and CD8^+^ T cells activated play a major role in anti-TB. As is well known, IFN-γ in peripheral blood is mainly produced by activated CD4^+^ effector T cells and a small amount of CD8^+^ effector T cells. IFN-γ, as a macrophage activating factor (MAF), induces the release of hydrogen peroxide (H_2_O_2_) and mediates antibacterial effects. In addition, IFN-γ, also as an immune modulator, mainly limits neutrophil recruitment and tissue inflammation-induced damage by inhibiting CD4^+^ T cell-mediated IL-17 generation (Saghazadeh and Rezaei, 2022). IFN-γ plays an important role in immune-mediated inhibition on *Mtb* infection. Our study found that the absolute counts of lymphocyte subsets in IGRA (+) TBM patients were higher than those in the IGRA (−) group, except for B cells (Yu Shan et al., 2021). The absolute counts of peripheral blood lymphocytes in IGRA (+) patients were mostly within the normal range, that is, the positive rate of IGRA in the patients with the absolute count of peripheral blood lymphocytes within the normal range was significantly higher than that in other patients (Xueqiong, 2020). These results demonstrated that the elevation of IFN-γ may be the key to host defense against TBM. Our study showed that the negative rate of IGRA in TBM patients was as high as 41.25% (33/80), significantly higher than that of PTB, and there was a trend of positive correlation between negative IGRA and the severity of TBM, which is consistent with the results of previous studies (Di and Li, 2018; Kim et al., 2018). It has been found that IGRA-negative TB patients had higher mortality, suggesting that IGRA-negative results may be a risk factor for the development of TBM, which may be caused by the reduction of CD4^+^ T cells and CD8^+^ T cells (Ngai et al., 2007; Nguyen et al., 2018; Yamasue et al., 2020). Multiple meta-analyses have shown that advanced age, low peripheral blood lymphocyte count, and decreased NKT cells may be common risk factors for false-negative IGRA (Nguyen et al., 2018; Yamasue et al., 2020), suggesting that cellular immune function is impaired in TBM patients (Wu Xueqiong, 2022). In addition, the absolute number of NKT cells in IGRA (+) TBM patients was significantly higher than that in the IGRA (−) group, which may be due to the proliferation of NKT cells to recognize glucolipid antigens after *Mtb* infection. It has been found that NKT cells were significantly higher in “rapid responders” (Kulpraneet et al., 2007; Pandey et al., 2019). Subsequently, activated NKT produces large amounts of IFN-γ and promotes the increase of other T lymphocyte subsets to combat *Mtb* invasion. Therefore, double detection of T lymphocyte subset absolute count and IGRAs in clinical practice may assist in identifying immunocompromised TB patients for timely immune intervention (Xu et al., 2018).

The humoral immune response of the body is mainly the specific antibody response generated by the differentiation of mature B lymphocytes induced by *Mtb* antigen into plasma cells, which plays a protective role in anti-TB immunity in collaboration with cellular immunity (Encinales et al., 2010; Achkar et al., 2015; Jacobs et al., 2016; Lu et al., 2016). Here we provide the first comparison of the changes in lymphocyte subsets in the peripheral blood of TBM patients with negative and positive antibodies. Our results showed that the absolute counts of all lymphocyte subsets in TBM patients in the Ab (−) group were higher than those in the Ab (+) group, suggesting that Th1-type protective immune response was dominant in TBM patients in the Ab (−) group, while T and B lymphocytes in the peripheral blood of TBM patients in the Ab (+) group were reduced, especially CD4^+^ T cells and B lymphocytes were significantly reduced, which may be caused by the migration of B cells to peripheral lymphoid organs, possibly indicating a decrease in the number of “protective” B cell subsets and an increase in potential pathological B cell subsets (Rijnink et al., 2021). After being stimulated by antigens, mature B cells migrated to peripheral lymphoid organs and became activated B cells, which then differentiate into plasma cells and increase the synthesis and secretion of antibodies, leading to the patient’s immune imbalance, with Th1-type immunity shifting toward Th2-type immunity, resulting in a weakened Th1-type immune response and an enhanced Th2-type immune response. This may be the reason why Ab (+) TBM patients have a positive correlation with disease severity. B cells not only produce antibodies, but also an antigen-presenting cell (APC) that produces pro-inflammatory and anti-inflammatory cytokines, such as IL-1β, IL-10, IL-17, IL-21, and TNF-α (du Plessis et al., 2016), which play an important role in the production and regulation of anti-TB immunity. In addition, we studied for the first time the changes in the number of peripheral blood immune cells in TBM patients at different BMRC stages. It was found that there was no significant difference in the lymphocyte absolute count between BMRC-stage I and stage II TBM patients, and the decrease was particularly obvious in BMRC-stage III TBM patients, and the proportion of the cases with NK cells and B cells below the normal reference value was significantly higher than that in BMRC-stage I and stage II patients. It is suggested that the decrease of NK cells may be one of the factors leading to the increase in the severity of clinical TBM patients, and the body’s immune response will also decrease, resulting in the decline of its resistance ability to *Mtb*, and the number of people with immune abnormalities will also increase (Holub et al., 2015; Thao et al., 2018). Thwaites et al. have found that among the HIV-negative TBM patients, the mortality was 20% in BMRC-stage I patients, 30% in BMRC-stage II patients, and 55% in BMRC-stage III patients (Tunkel, 2005). Therefore, in the early diagnosis and clinical treatment monitoring process of TBM patients, when the absolute count of various lymphocyte subsets in patients plummeted, especially NK cell changes, it is necessary to timely judge the disease progression to adjust the clinical treatment regimen, reduce mortality, prevent disease deterioration, and improve prognosis.

The main limitations of this study are as follows: (1) Due to the low incidence of TBM, the sample size of this study is not large enough, especially for severe TBM patients with BMRC-stage III; (2) This study focused on the number and function of immune cells in peripheral blood, without simultaneously studying immune cells in CSF. Therefore, we will further study the distribution and functional changes of immune cells, especially NK cells, in CSF of TBM patients in future studies.

## 5 Conclusion

In conclusion, the absolute counts of lymphocyte subsets, especially NK cells, in the peripheral blood of TBM patients were significantly reduced. Moreover, the reduction of these immune cells was closely related to the disease severity and had a certain correlation with cellular and humoral immune responses. Therefore, this study understood the clinical immunological characteristics of TBM patients, which helps to better understand the immune mechanism of TBM caused by *Mtb* infection. It can provide reliable indicators for evaluating the immune status of TBM patients in clinical practice and provide the experimental basis for future host-directed therapy of TBM patients.

## Data availability statement

The original contributions presented in the study are included in the article/supplementary material, further inquiries can be directed to the corresponding authors.

## Ethics statement

The studies involving humans were approved by the Ethics Committee of the 8th Medical Center of the PLA General Hospital (approval number: 309201904081530). The studies were conducted in accordance with the local legislation and institutional requirements. The participants provided their written informed consent to participate in this study.

## Author contributions

JM: Data curation, Formal Analysis, Writing – original draft. YiL: Data curation, Validation, Writing – original draft. YX: Data curation, Validation, Writing – original draft. WS: Supervision, Writing – original draft. YaL: Supervision, Writing – original draft. HA: Writing – review & editing. XW: Conceptualization, Methodology, Project administration, Supervision, Writing – review & editing. JL: Conceptualization, Writing – review & editing.
