# Supplementary material for: The changes and its significance of peripheral blood NK cells in patients with tuberculous meningitis
Source: Front Microbiol. 2024 Feb 29;15:1344162. doi: 10.3389/fmicb.2024.1344162 (PMC10937341; doi:10.3389/fmicb.2024.1344162)
Supplement: Supplementary file 1 [file Table_1.DOCX]

**Supplementary Table 1 The frequency of lymphocyte subsets in each group**

|  |  | **Frequency % of lymphocyte subsets** | | | | | | |
| --- | --- | --- | --- | --- | --- | --- | --- | --- |
| **Group** | **n** | **CD3^+^ T** | **CD4^+^ T** | **CD8^+^ T** | **CD4^+^/CD8^+^** | **NK** | **NKT** | **B** |
| **NC** | 96 | 70.12 | 34.57 | 26.65 | 1.28 | 17.49 | 5.82 | 10.72 |
| **LTBI** | 50 | 67.23 | 38.70 | 25.12 | 1.56 ^a^ | 17.48 | 6.15 | 12.29 |
| **NSTB** | 125 | 72.94 ^b^ | 41.72 ^a^ | 24.70 | 1.55 ^a^ | 11.76 ^ab^ | 5.74 | 11.46 |
| **TBM** | 119 | 74.47 ^ab^ | 41.32 ^a^ | 26.94 | 1.47 | 10.04 ^ab^ | 5.44 | 11.29 |
| **Simple TBM** | 40 | 74.44 | 43.87 | 25.13 | 1.68 | 8.09 | 4.72 | 13.21 |
| **TBM-ATB** | 79 | 74.47 | 40.79 | 27.74 | 1.39 | 10.86 | 5.78 | 9.88 |
| **Treatment Duration** |  |  |  |  |  |  |  |  |
| **In-TBM** | 37 | 75.47 | 39.64 | 26.41 | 1.40 | 9.94 | 6.91 | 12.48 |
| **Re-TBM** | 82 | 74.18 | 41.53 | 27.36 | 1.49 | 10.14 | 5.20 | 10.32 |
| **BMRC** |  |  |  |  |  |  |  |  |
| **Stage I** | 87 | 73.88 | 40.79 | 26.98 | 1.39 | 10.04 | 5.90 | 10.11 |
| **Stage II** | 22 | 70.99 | 43.41 | 26.88 | 1.77 | 10.17 | 4.21 | 13.04 |
| **Stage III** | 10 | 77.58 | 42.23 | 28.37 | 1.49 | 9.56 | 7.56 | 9.93 |
| **CSF/Sputum** |  |  |  |  |  |  |  |  |
| **EG (-)** | 58 | 77.11 | 43.04 | 27.07 | 1.60 | 8.70 | 5.84 | 10.66 |
| **EG (+)** | 17 | 73.88 | 41.32 | 27.06 | 1.50 | 10.08 | 4.56 | 9.70 |
| **Cellular Immune** |  |  |  |  |  |  |  |  |
| **IGRA (-)** | 33 | 72.56 | 42.07 | 26.23 | 1.59 | 10.40 | 4.19 | 12.53 |
| **IGRA (+)** | 57 | 73.69 | 41.87 | 26.82 | 1.56 | 9.94 | 5.79 | 10.72 |
| **Humoral Immune** |  |  |  |  |  |  |  |  |
| **AB (-)** | 39 | 72.60 | 43.30 | 25.28 | 1.66 | 9.72 | 4.66 | 12.79 |
| **AB (+)** | 46 | 71.05 | 41.09 | 26.72 | 1.47 | 10.63 | 5.65 | 10.17 |

^a^ *P*<0.05 *vs* NC; ^b^ *P*<0.05 *vs* LTBI
