# Supplementary material for: The changes and its significance of peripheral blood NK cells in patients with tuberculous meningitis
Source: Front Microbiol. 2024 Feb 29;15:1344162. doi: 10.3389/fmicb.2024.1344162 (PMC10937341; doi:10.3389/fmicb.2024.1344162)
Supplement: Supplementary file 2 [file Table_2.DOCX]

**Supplementary Table 2 The relationship between the incidence of IGRA (-) and Ab (+) in TBM patients and the disease severity**

|  | **BMRC** **case number (%)** | | |
| --- | --- | --- | --- |
|  | **Stage I** | **Stage II** | **Stage III** |
| **Cellular Immune** |  |  |  |
| **IGRA (-)** | 22 (25.3%) | 7 (31.8%) | 4 (40.0%) |
| **IGRA (+)** | 40 (46.0%) | 12 (54.5%) | 6 (60.0%) |
| **Humoral Immune** |  |  |  |
| **Ab (-)** | 29 (33.3%) | 7 (31.8%) | 3 (30.0%) |
| **Ab (+)** | 31 (35.6%) | 10 (45.5%) | 5 (50.0%) |
